# Intralayer Phonons in Multilayer Graphene Moiré Superlattices

**DOI:** 10.34133/2022/9819373

**Published:** 2022-05-30

**Authors:** Miao-Ling Lin, Min Feng, Jiang-Bin Wu, Fei-Rong Ran, Tao Chen, Wei-Xia Luo, Heng Wu, Wen-Peng Han, Xin Zhang, Xue-Lu Liu, Yang Xu, Hai Li, Yu-Fang Wang, Ping-Heng Tan

**Affiliations:** ^1^State Key Laboratory of Superlattices and Microstructures, Institute of Semiconductors, Chinese Academy of Sciences, Beijing 100083, China; ^2^Center of Materials Science and Optoelectronics Engineering & CAS Center of Excellence in Topological Quantum Computation, University of Chinese Academy of Sciences, Beijing 100049, China; ^3^School of Physics, Nankai University, Tianjin 300071, China; ^4^Key Laboratory of Flexible Electronics and Institute of Advanced Materials, Jiangsu National Synergetic Innovation Center for Advanced Materials, Nanjing Tech University, 30 South Puzhu Road, Nanjing 211816, China; ^5^School of Micro-nanoelectronics, State Key Laboratory of Silicon Materials, Hangzhou Global Scientific and Technological Innovation Center, ZJU-UIUC Joint Institute, Zhejiang University, Hangzhou 310027, China

## Abstract

Moiré pattern in twisted multilayers (tMLs) induces many emergent phenomena by subtle variation of atomic registry to modulate quasiparticles and their interactions, such as superconductivity, moiré excitons, and moiré phonons. The periodic superlattice potential introduced by moiré pattern also underlies patterned interlayer coupling at the interface of tMLs. Although this arising patterned interfacial coupling is much weaker than in-plane atomic interactions, it is crucial in moiré systems, as captured by the renormalized interlayer phonons in twisted bilayer transitional metal dichalcogenides. Here, we determine the quantitative relationship between the lattice dynamics of intralayer out-of-plane optical (ZO) phonons and patterned interfacial coupling in multilayer graphene moiré superlattices (MLG-MS) by the proposed perturbation model, which is previously challenging for MLGs due to their out-of-phase displacements of adjacent atoms in one atomic plane. We unveil that patterned interfacial coupling introduces profound modulations on Davydov components of nonfolded ZO phonon that are localized within the AB-stacked constituents, while the coupling results in layer-extended vibrations with symmetry of moiré pattern for moiré ZO phonons. Our work brings further degrees of freedom to engineer moiré physics according to the modulations imprinted on the phonon frequency and wavefunction.

## 1. Introduction

The misorientation or lattice mismatch between the constituents of van der Waals (vdW) heterostructures (vdWHs) enables the generation of moiré superlattices, which impose nanoscale periodic modulations on the electronic band [1, 2] and optical selection rules [3], and lead to correlated electronic phases [4, 5], superconductivity [6, 7], and moiré excitons [3, 8–10]. Particularly, the interfacial coupling is also modulated by the nanopatterned periodic potential of moiré superlattices, leading to patterned interfacial coupling (PIC) and thus a platform of versatile phonon tunability [11, 12]. Recently, the roles of phonons in many emerging quantum phenomena of vdWHs have been emphasized [13–16]. The control of phonon and its interaction with electronic phases in moiré structures is considered as potential key point for many exotic properties absent in the constituents, such as insulator-metal transition and ferromagnetism [11, 12, 17].

In the light of the above long-pursued goals, the modulation of moiré superlattices and the corresponding PIC on phonon properties have attracted much attention [11, 12, 18, 19]. Previous studies have identified moiré phonons in twisted bilayer transitional metal dichalcogenides (TMDs) [18] that are activated by phonon folding effect in moiré superlattices, whose vibrations are engineered by atomic registry. Meanwhile, the interlayer phonons in reconstructed moiré superlattices of twisted bilayer MoS_2_ are renormalized owing to the strong coupling with moiré acoustic phonons [12]. However, in moiré superlattices of graphene or hexagonal boron nitride (hBN) whose monolayer is comprised of only one atomic plane, the imprints of PIC on their intralayer phonons are inherently complex because the displacements of adjacent atoms in one atomic plane are out-of-phase, and those in the adjacent atomic planes vary from local to local due to twist-angle dependent interlayer registries. Recent nano-Raman spectroscopy [11] and nanoinfrared imaging [19] revealed localized lattice dynamics of nonfolded in-plane intralayer phonons in low-angle twisted bilayer graphene (tBLG) and twisted hBN, respectively. In large-angle moiré superlattices beyond the spatial limit of nanotechnologies, the evolution of intralayer phonons upon modulation of PIC remains appealing, especially in multilayer graphene (MLG) moiré superlattices (MLG-MS) with additional and tunable degrees of freedom in quantum manipulation and device characteristics [5, 20–22] owing to the coexistence of PIC and natural interlayer coupling. And two general open questions stand out: (a) How do moiré superlattices modulate the lattice dynamics of intralayer phonons? (b) How does PIC affect the vibrations related to intralayer phonons in the graphene layers that are not adjacent to the moiré interface?

Herein, we reveal the modulation of PIC on the frequency and atomic displacements of intralayer phonons by the resonance Raman spectroscopy of MLG-MS containing both twisted and AB-stacked interfaces. This is manifested in the renormalized Davydov splitting for both intralayer out-of-plane optical (ZO) phonons at the unfolded Brillouin zone (BZ) center, *i.e.*, nonfolded ZO phonons and moiré ZO (mZO) phonons folded from the off-center phonons of untwisted constituents. The renormalized Davydov components of nonfolded ZO phonons are localized within the AB-stacked constituents due to the zero perturbation from PIC, which is rationalized by a perturbation model (PM) and confirmed by the force constant method (FCM). However, the PIC results in vibrations extended to all the stacking layers for the Davydov components of mZO phonons, where the vibration pattern shows similar symmetry to moiré superlattices. This work enhances the understanding about the effect of PIC on phonons in moiré superlattices, pointing to potential opportunities for phonon manipulation.

## 2. Results

### 2.1. Enhanced Davydov Components of ZO Modes in MLG-MS

**Figure 1:**
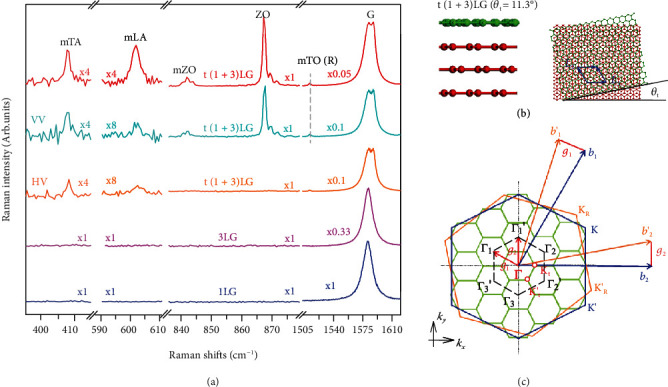
Raman spectra and the schematic of BZ in t(1 + 3)LG. (a) Raman spectroscopy of t(1 + 3) LG with a twist angle (*θ*_t_) of 11.3° and its constituents excited by 1.96 eV. Spectra in parallel (VV) and crossed (HV) configurations are also shown. (b) Schematic structure of t(1 + 3) LG where the 1LG (green) sits on the top of the 3LG (red) with *θ*_t_ = 11.3°. Vectors ***L_1_*** and ***L_2_*** define the supercell. (c) The reciprocal lattice of t(1 + 3)LG. **b**_1_(**b**′_1_) and **b**_2_(**b**′_2_) are the reciprocal vectors of the bottom 3LG (top 1LG), whereas **g**_1_ and **g**_2_ are those of t(1 + 3)LG. Small hexagons represent the reciprocal Wigner-Seitz cells of moiré superlattices in t(1 + 3)LG.

Moiré phonons linked with the reciprocal unit vectors of the moiré superlattices within the BZ interior of untwisted constituents can be activated by the phonon folding effect in the Raman spectroscopy of twisted bilayers [18, 23–27], where rich resonance mechanisms of moiré phonon modes were reported [18, 26, 28]. Similar phenomena are also expected in MLG-MS, which are assembled by *m*-layer graphene (*m*LG) and *n*LG and are denoted as t(*m* + *n*)LG (See Methods, Supplementary Figure S1 and Figure S2). If *m*(*n*) > 1, *m*LG (*n*LG) is AB-stacked. The Raman spectrum of t(1 + 3)LG in Figure 1(a) is resonantly excited by 1.96 eV, and its G mode intensity (*I*(G)) is ~20 times stronger than that of 1LG, similar to the giant enhancement of G mode in tBLG [28–31]. Closer inspection shows that the G band exhibits obvious doubling, which is common in t(*m* + *n*)LG (*m* or *n*>1) and is connected to a resonant process determined by twist angle [30]. The relative Raman intensity between the two subpeaks is sensitive to the excitation energy. The characteristic R peak [23] below the G mode originates from the moiré phonon associated with the transverse optical (TO) phonon branch, which is denoted as mTO phonon here. This peak is observed at ~1510 cm^−1^, which corresponds to a twist angle (*θ*_t_) of 11.3° based on the *θ*_t_-dependent mTO phonon frequency [26, 32]. Figures 1(b) and 1(c) show the stacking schematics of t(1 + 3)LG with *θ*_t_ = 11.3° (also in Supplementary Figure S3) and the corresponding reciprocal lattices, respectively. The lengths of basic wave vectors of moiré superlattices with *θ*_t_ are defined by $\left|g_{i}\left(\theta_{\text{t}}\right)\right|=8\pi /\sqrt{3}a\sin\left(\theta_{\text{t}}/2\right)$ and $a=2.46\;\overset{{}^{\circ}}{\text{A}}$ [18, 23]. The phonons at Γ_*j*_/Γ′_*j*_ (*j* = 1, 2, 3) points determined by **g**_*i*_ within the BZ of the constituents, *i*.*e*. moiré phonons, are folded back to the BZ center of moiré superlattices. Because t(1 + 3)LG shows *C*_3_ symmetry [33], its in-plane and out-of-plane vibrations become Raman-active and are assigned to *E* and *A* irreducible representations, respectively. Thus, the moiré phonons are expected to be observed in Raman spectra. Besides the mTO or R mode, additional Raman peaks are also observed in t(1 + 3)LG in the spectral range of 400–900 cm^−1^, when compared with those in 1LG and 3LG. Based on the phonon dispersion [34] and the deduced *θ*_t_, the extra peaks in t(1 + 3)LG at ~408 cm^−1^ and ~602 cm^−1^ are assigned to the moiré phonons related to transverse and longitudinal acoustic phonon branches, and they are denoted as mTA and mLA, respectively. Furthermore, the modes at ~844 cm^−1^ and ~870 cm^−1^ are, respectively, moiré (*i.e.*, mZO) phonon and nonfolded phonons linked with the ZO phonon branch. The mTA and mLA modes are observed in parallel (VV) and crossed (HV) configurations, whereas mZO and nonfolded ZO phonons appear only in the VV configuration. This further confirms the above assignments. In contrast to one Raman peak related to each moiré phonon and no feature linked with nonfolded ZO mode in tBLG [26, 32], three peaks with a width of ~1 cm^−1^ each are clearly resolved for both mZO and nonfolded ZO modes in t(1 + 3)LG.

Fundamentally, the optical modes in *N*LG undergo Davydov splitting due to the weak interlayer coupling and split up into *N* Davydov components [35]. For example, the silent ZO mode (*B*_2*g*_) in 1LG is expected to split up into two silent *A*′′_2_ and one Raman-active *A*′_1_ modes for 3LG, although none of them are observed in Raman spectroscopy due to Raman-inactivity or weak electron-phonon coupling. Considering the comparable interfacial and interlayer breathing coupling in MLG-MS [33], four Davydov components are expected for ZO phonons in t(1 + 3)LG, inconsistent with the three Davydov-split peaks of nonfolded ZO and mZO modes observed in experiments. Notably, the mZO and ZO phonon modes can only be resonantly detected in a narrow excitation energy range.

**Figure 2:**
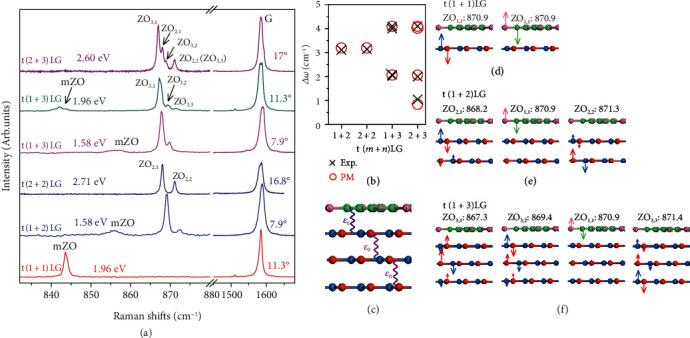
Davydov splitting of nonfolded ZO modes in t(*m* + *n*)LGs and PM. (a) Raman spectra for t(*m* + *n*)LGs. (b) The experimental (crosses) and calculated (circles) frequency differences (Δ*ω*) between each Davydov component and the lowest-frequency one in t(*m* + *n*)LGs. (c) Schematic of PM for t(1 + 3)LG involving perturbation from interlayer (*ε*_0_) and interfacial (*ε*_t_) coupling. Atomic displacements and mode frequencies (in cm^−1^) of Davydov components for nonfolded ZO phonons in (d) t(1 + 1)LG, (e) t(1 + 2)LG, and (f) t(1 + 3)LG calculated by the PM. Arrow lengths represent vibration amplitudes.

To understand the observed Davydov splitting of the nonfolded ZO and mZO phonons in MLG-MS and their modulation by interlayer coupling at AB-stacked and twisted interfaces, we measure the Raman spectra of other t(*m* + *n*)LGs under the resonant excitation conditions, as plotted in Figure 2(a). As the commonly used laser in the visible region cannot match the van Hove singularities in electronic joint density of states of all the optically allowed transitions in t(*m* + *n*)LGs  with *θ*_t_ close to 0° and 30° [26], in this work, we only present the results in t(*m* + *n*)LGs with 7.9° ≤ *θ*_t_ ≤ 18°. Similar to t(1 + 3)LG (Figure 1(a)), the G band in t(*m* + *n*)LGs (*m* or *n* > 1) consists of two subbands. The Raman peaks at ~870 cm^−1^ originate from nonfolded ZO phonon, while the modes with peak positions varying with samples are mZO phonon modes, e.g., peaks at ~844 cm^−1^ in t(1 + 1)LG and t(1 + 3)LG with *θ*_t_ = 11.3° and peaks at ~855 cm^−1^ in t(1 + 2)LG and t(1 + 3)LG with *θ*_t_ = 7.9°. Except for t(1 + 1)LG, the nonfolded ZO and mZO modes in t(*m* + *n*)LGs are both activated and exhibit Davydov splitting, although *I*(mZO) is much weaker than *I*(ZO). Interestingly, the number of observed Davydov components of nonfolded ZO modes in t(*m* + *n*)LG is directly related to the number of layers of its constituents, but not equal to its total number (*i.e.*, *N* = *m* + *n*). For example, we observe three and two Davydov components of nonfolded ZO modes in t(1 + 3)LG and t(2 + 2)LG, respectively, rather than four Davydov components. This suggests that the interfacial coupling in t(*m* + *n*)LG is too weak to induce *m* + *n* Davydov components of nonfolded ZO phonons. Furthermore, the frequency difference between the two adjacent Davydov components stays constant (~2.0 cm^−1^) in t(1 + 3)LGs with different *θ*_t_ (Figure 2(b)).

### 2.2. Localized Davydov Components of Nonfolded ZO Modes

Davydov splitting of intralayer phonons induced by interlayer coupling in multilayer and twisted TMDs is well represented by the vdW model [36–38], where the interlayer coupling can be treated as an overall force due to the in-phase atomic displacements in one atomic plane. The frequency differences (Δ*ω*) between each Davydov component and the lowest-frequency one are closely related to the frequency of layer-breathing (LB) phonons. We tried to extend this vdW model to the nonfolded ZO phonons in t(*m* + *n*)LGs both with and without interfacial LB coupling considered (Supplementary Figure S4), while obvious discrepancy is present between the calculated and experimental results. This suggests that the vdW model is not valid in understanding the Davydov splitting of nonfolded ZO phonons in MLG-MS, where the adjacent atoms in one atomic plane are out-of-phase and interlayer coupling cannot be assumed as a residual restoring force to all atoms. Looking for insights into the Davydov splitting of nonfolded ZO phonons, the Davydov components are derived from the linear superpositions of ZO phonons in each graphene layer. The influence from weak interlayer coupling can be assumed as a perturbation to such equivalent ZO phonons, which is not only associated with periodic potential of PIC but also the atomic displacements of nonfolded ZO phonon. In this case, we develop a model from perspective of perturbation to understand the Davydov splitting of nonfolded ZO phonons in t(*m* + *n*)LG, where the unperturbed ZO_1,1_ mode in 1LG is treated as the basis, and the perturbation from interlayer (interfacial) coupling is *ε*_0_ (*ε*_t_), as schematized in Figure 2(c).

Taking t(1 + 2)LG as an example, the reduced Hamiltonian is

(1) $$\begin{array}{c} H_{\text{t}\left(1+2\right)\text{LG}}=E_{0}I+\left(\begin{array}{ccc} 0 & \varepsilon_{\text{t}} & 0\\ \varepsilon_{\text{t}} & \delta & \varepsilon_{0}\\ 0 & \varepsilon_{0} & 0 \end{array}\right)=\left(\begin{array}{ccc} E_{0} & \varepsilon_{\text{t}} & 0\\ \varepsilon_{\text{t}} & E_{0}+\delta & \varepsilon_{0}\\ 0 & \varepsilon_{0} & E_{0} \end{array}\right), \end{array}$$

where *E*_0_ is the energy of an unperturbed ZO phonon, *i.e.*, ZO_1,1_ in 1LG. *E*_0_ is set as 870.9 cm^−1^ to match the nonfolded ZO mode frequencies to the experimental ones in t(1 + 2)LG and other t(*m* + *n*)LGs. *δ* is introduced to follow the change of the on-site energy in the middle layer due to the new geometry, similar to the on-site energy variation due to environmental changes in the electronic tight-binding model [39]. Considering that the number of observed Davydov components of nonfolded ZO modes in t(*m* + *n*)LG is related to *m* and *n* rather than *N* = *m* + *n* (Figure 2(a)), the impact of interfacial coupling on the nonfolded ZO modes is negligible. Thus, we assume *ε*_t_ = 0, and then, we solve the secular equation det(*H* − *λI*) = 0. According to the observed splitting frequency of 3.16 cm^−1^ in t(1 + 2)LG, |*ε*_0_| and |*δ*| are deduced as 1.1 cm^−1^ and 2.3 cm^−1^, respectively. We find the positive *ε*_0_ and negative *δ* have reasonable physical significance and can reproduce all the experimental results, as discussed below. The corresponding eigenvectors are *ϕ*_1_ = (1, 0, 0)^T^, *ϕ*_2_ = (0,−0.93, 0.37)^T^, and *ϕ*_3_ = (0,−0.37,−0.93)^T^. The vibration corresponding to *ϕ*_1_ is localized in 1LG (ZO_1,1_), which cannot be observed due to its Raman inactivity in 1LG. The latter two eigenvectors correspond to the two observed Davydov components in t(1 + 2)LG, *i.e.*, ZO_2,*i*_ (*i* = 1, 2), and frequency increases with *i*.  ZO_2,1_ and ZO_2,2_ modes are associated with the out-of-phase and in-phase superpositions of the two unperturbed ZO modes, respectively.

Similar analysis with *ε*_t_ = 0 can be extended to understand the Davydov splitting of nonfolded ZO phonon in other t(*m* + *n*)LGs. The corresponding calculated eigenvectors (*i.e.*, atomic displacements) are shown in Figures 2(d)–2(f)). In t(1 + 1)LG, two equivalent ZO_1,1_ modes with their vibrations localized in the top or bottom 1LG are predicted by the PM. Thus, no ZO mode is activated in t(1 + 1)LG, in line with the experimental result (Figure 2(a)). In addition, there are two Davydov components of nonfolded ZO phonons in t(2 + 2)LG, each doubly degenerate (Supplementary Figure S5). For t(1 + 3)LG, four eigenvectors are obtained, among which ZO_1,1_ (*ϕ*_1_ = (1, 0, 0, 0)^T^) localizes in the 1LG constituent and the other three localize within the 3LG constituents. With the abovedetermined values of *ε*_0_ (1.1 cm^−1^) and *δ* (-2.3 cm^−1^), Δ*ω* between ZO_3,2_ and ZO_3,1_ components and between ZO_3,3_ and ZO_3,1_ components are, respectively, 2.1 cm^−1^ and 4.1 cm^−1^, which agree well with the experimental data, as elucidated in Figure 2(b). In particular, the PM also reproduces the five Davydov components of nonfolded ZO modes in t(2 + 3)LGs, among which the two highest-frequency components exhibit almost the same frequency. In this case, four Davydov components are resolved in Raman spectroscopy. These results indicate the validity of PM to understand the localized Davydov components of nonfolded ZO phonons within the constituents of t(*m* + *n*)LGs, including the assumption of negligible perturbation from PIC (*i.e.*, *ε*_t_ = 0). Only one *δ* is used in the PM for the middle layers in MLG-MS, no matter they are adjacent to the twisted or AB-stacked interface. Notably, *ε*_t_ and *δ* are closely linked with both the PIC and atomic displacements of nonfolded ZO phonon in one atomic plane. Although the moiré pattern in MLG-MS largely reduces the perturbation from interfacial coupling on nonfolded ZO phonons due to the planar locally mismatched periodicity of the charge density variation [33], it keeps the on-site energy variation *δ* in the two interfacial layers of MLG-MS the same as that in AB stacking, which may be ascribed to the comparable LB coupling at AB-stacked and twisted interfaces. Thus, the PIC in MLG-MS significantly modulates the lattice dynamics of nonfolded ZO phonons.

### 2.3. Renormalized Davydov Components of Nonfolded ZO Modes by PIC

**Figure 3:**
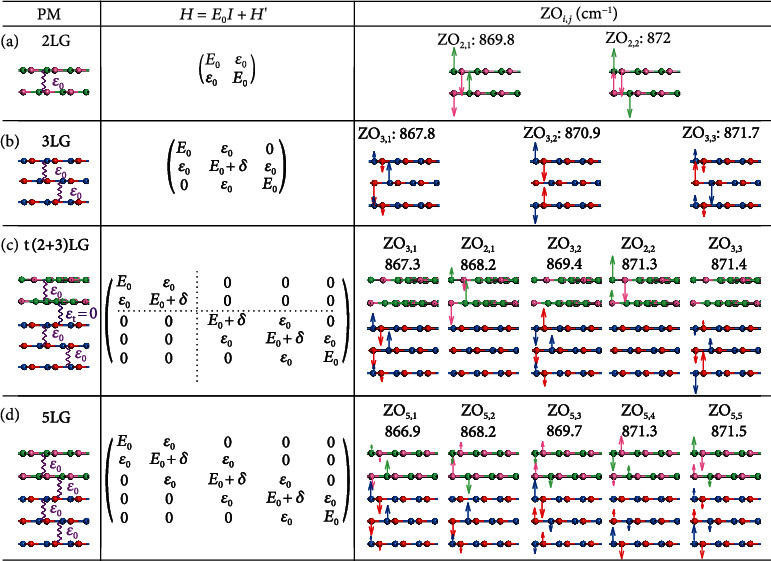
PM in MLG and t(*m* + *n*)LGs. Schematic of PM and reduced Hamiltonian and atomic displacements of Davydov components in (a) 2LG, (b) 3LG, (c) t(2 + 3)LG, and (d) 5LG.

In principle, the PM with the abovedetermined values of *ε*_0_ and *δ* is also applicable to estimate the Davydov components of ZO phonons in AB-stacked MLG. For example, two Davydov components at 869.8 cm^−1^ and 872 cm^−1^ are predicted in 2LG (Figure 3(a)), while three Davydov components are anticipated in 3LG (Figure 3(b)). One would intuitively expect that the Davydov splitting in t(*m* + *n*)LG is the same as those in AB-stacked *m*LG and *n*LG due to the negligible effect of interfacial coupling on nonfolded ZO phonons. However, the case is different due to the on-site energy variation, as exemplified by the comparison between 2LG, 3LG, and t(2 + 3)LG. In t(2 + 3)LG (Figure 3(c)), the reduced Hamiltonian can be divided into two nonzero submatrices (as divided by the dotted lines) related to the 2LG and 3LG constituents due to *ε*_t_ = 0, whereas these two submatrices are different from those in pristine 2LG and 3LG due to the presence of *δ* in the interfacial layers of the 2LG and 3LG constituents. Thus, the resulting frequencies and atomic displacements of Davydov components related to nonfolded ZO phonon in t(2 + 3)LG (Figure 3(c)) are distinguished from those in pristine 2LG (Figure 3(a)) and 3LG (Figure 3(b)). In addition, when the top 2LG and bottom 3LG are assembled in AB stacking at the twisted interface and a 5LG is formed, the perturbation from the interfacial coupling is *ε*_0_. Thus, the ZO phonon splits up into five Davydov components, which stems from the vibrations of the five stacking layers, as elucidated in Figure 3(d). Their frequencies also show obvious distinction from those in t(2 + 3)LG. These contrasts imply profound modulations of the PIC on the lattice dynamics of nonfolded ZO phonons in MLG-MS.

**Figure 4:**
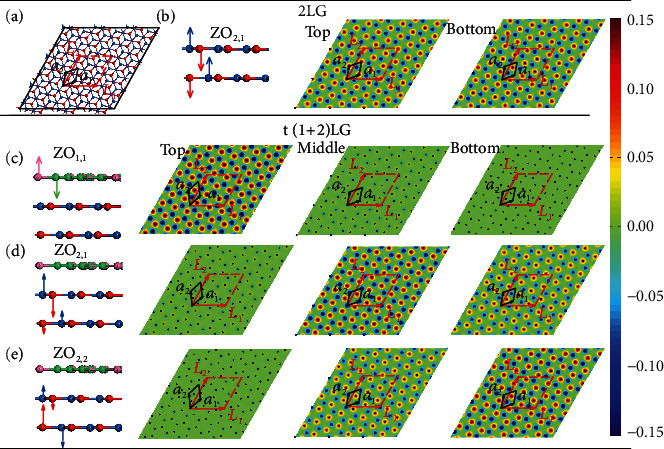
Atomic displacements of Davydov components of nonfolded ZO modes based on FCM. (a) A supercell and (b) atomic displacements of the ZO_2,1_ mode in 2LG. **a*_1_*** and **a*_2_*** are the lattice vectors of the unit cell in graphene, and ***L_1_*** and ***L_2_*** correspond to the lattice vectors of the moiré unit cell in t(1 + 2)LG with *θ*_t_ = 21.8°. Atomic displacements of (c) ZO_1,1_, (d) ZO_2,1_, and (e) ZO_2,2_. Color codes are based on the amplitudes of atomic displacements.

More imprints for the PIC on nonfolded ZO phonons can be found in the atomic displacements calculated by the FCM by considering the Born-von Karman model with the interactions along the radial and tangential directions (see Materials and Methods) [40, 41]. For calculation simplicity, we take t(1 + 2)LG with *θ*_t_ = 21.8° as an example. A supercell of the atomic structure for 2LG and the vibration patterns of ZO_2,1_ are plotted (Figures 4(a) and 4(b)). ZO_2,2_ in 2LG exhibits similar atomic displacements to ZO_2,1_ except for in-phase superposition of the two nonfolded ZO phonons in each layer. For t(1 + 2)LG, the calculation presents two Davydov components in the 2LG constituent due to the interlayer coupling and one ZO_1,1_ mode with vibration localized in the 1LG constituent, as shown in Figures 4(c)–4(e)). This confirms that the interfacial coupling is too weak to couple the three graphene layers for nonfolded ZO phonons. Additionally, for the ZO_2,1_ and ZO_2,2_ modes in t(1 + 2)LG, the vibration amplitude ratios of the superposition atoms between the two bottom layers are 1.5:(−1) and 1 : 1.5, respectively, distinct from those in pristine 2LG (Figure 4(b)). This underpins the modulation of moiré superlattices on atomic displacements of nonfolded ZO phonons in MLG-MS. The above analysis based on the PM and FCM is universally valid to estimate the Davydov components of intralayer phonons and the effects of interlayer/interfacial coupling on them in other vdWHs.

**Figure 5:**
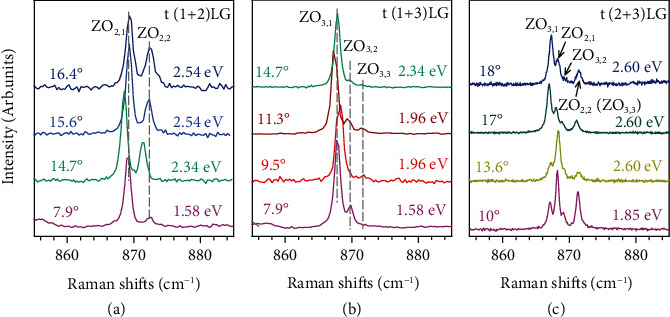
Raman spectra of t(*m* + *n*)LGs. Raman spectra of nonfolded ZO modes in (a) t(1 + 2) LGs, (b) t(1 + 3)LGs, and t(2 + 3)LGs with different twist angles, which are under specific resonance excitations.

We notice that the localized and renormalized Davydov components linked with the nonfolded ZO phonon are ubiquitously observed in MLG-MS under resonance excitation, as demonstrated by the Raman spectra of t(1 + 2)LGs, t(1 + 3)LGs, and t(2 + 3)LGs with various *θ*_t_ in Figure 5. For t(1 + 2)LGs, ZO_2,1_ and ZO_2,2_ components with a splitting frequency of ~3.0 ± 0.15 cm^−1^ are present. As for t(1 + 3)LGs, three peaks (*i.e.*, ZO_3,1_, ZO_3,2_, and ZO_3,3_ components) are observed when *θ*_t_ = 11.3° and 14.7°, while two distinct Davydov components are observed when *θ*_t_ = 7.9° and 9.5°, whose splitting frequency is quite close to the estimated Δ*ω* between ZO_3,2_ and ZO_3,1_ components and between ZO_3,3_ and ZO_3,1_ components by PM, respectively. Four Davydov components are observed in t(2 + 3)LGs, where each frequency difference between two given Davydov components varies slightly with twist angle. Despite the Davydov splitting in MLG-MS is independent of *θ*_t_, the resonant excitation energy and the relative Raman intensity of Davydov components are closely associated with *θ*_t_, pointing to the twist-angle dependent electronic transition and electron-phonon coupling. It is also worth noting that the reduced symmetry by twisting and resonance excitation are prerequisites for the observations of Davydov components of ZO phonons in MLG-MS.

### 2.4. Layer-Extended Davydov Components of mZO Modes Modulated by Moiré Pattern

**Figure 6:**
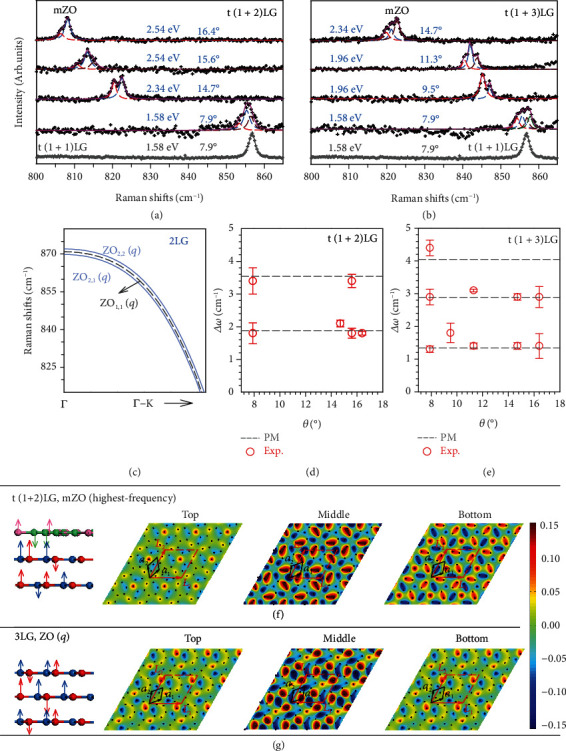
Davydov components of mZO modes in t(*m* + *n*)LG. Raman spectra of mZO modes in (a) t(1 + 2)LGs and (b) t(1 + 3)LGs in comparison with t(1 + 1)LG. (c) The dispersion of ZO phonon branch in 2LG and 1LG. The experimental (Exp.) frequency differences (open circles) between each Davydov component and the lowest-frequency one (Δ*ω*) of mZO modes in (d) t(1 + 2)LGs and (e) t(1 + 3)LGs, along with the calculated results from PM (dashed lines). Atomic displacements of (f) the highest-frequency mZO in t(1 + 2)LG and (g) the corresponding unfolded ZO(*q*) in 3LG. Color codes are based on the amplitudes of atomic displacements.

We further look for insights into the effects of the PIC in moiré pattern on the mZO phonons. The maximum number of observed Davydov components linked with mZO phonons in t(*m* + *n*)LG can be up to *m* + *n*. For specific t(*m* + *n*)LG, the number of observed Davydov components is dependent on *θ*_t_ and laser excitation, as shown in Figures 6(a) and 6(b). We first assume that the observed splitting peaks in t(*m* + *n*)LG only correspond to Davydov components of mZO phonon in its constituents due to the weak impact from interfacial coupling. Following this hypothesis and the phonon dispersion calculated by FCM (Figure 6(c)), we should observe three peaks of mZO phonons in a t(1 + 2)LG, in which the one corresponding to the mZO phonon from 1LG constituent shows frequency between the two Davydov components of mZO phonons in 2LG. However, by comparing the mZO peaks in t(1 + 2)LG with t(1 + 1)LG at the same *θ*_t_ of ~7.9° in Figure 6(a), we find that the frequency of the mZO peak in t(1 + 1)LG approaches the highest-frequency components of mZO mode in t(1 + 2)LG. This implies the frequencies of the Davydov components of mZO peaks in MLG-MS are altered by the interfacial coupling. When providing further insights into the frequency differences between each Davydov entity and the lowest-frequency one, as shown in Figures 6(d) and 6(e), we find that each Δ*ω* stays constant for t(1 + 2)LGs with *θ*_t_ ranging from 7.9 ° to 16.4 °. And it is also the case in t(1 + 3)LGs. This indicates that the perturbations from the interlayer and interfacial couplings vary slightly with large *θ*_t_ in MLG-MS. We also extended the proposed PM to understand the Davydov components of mZO phonons in t(*m* + *n*)LGs and to estimate the perturbations from interlayer and interfacial couplings. We found the calculated results (dashed lines) based on PM roughly agree with the experimental results when |*ε*_0_| = |*ε*_t_| = 1.25 cm^−1^ and *δ* = −0.2 cm^−1^. Hence, the perturbations from interlayer coupling and PIC on mZO phonons in t(*m* + *n*)LG are equivalent; thus, the atomic displacements of mZO Davydov components extend to all the stacking layers in MLG-MS. This is distinct from the case of nonfolded ZO phonons.

Clear signatures of the modulation from the moiré pattern on the mZO Davydov components are found in the atomic displacements calculated by the FCM, as demonstrated in Figure 6(f) (also in Supplementary Figure S6 and Figure S7). The calculations demonstrate that the mZO Davydov components stem from the vibrations of the three stacking graphene layers in t(1 + 2)LG, in consistent with the predictions from PM. More interestingly, the vibration patterns at each layer show *C*_3_ symmetry. However, the corresponding unfolded ZO(*q*) phonons in 3LG exhibit distinctly different vibration patterns, without any signature of *C*_3_ symmetry, as shown in Figure 6(g). This further confirms the crucial role of the moiré pattern in the modulation for both frequency and lattice dynamics of mZO phonons in MLG-MS. The excitation-energy dependent mZO Davydov components observed in t(*m* + *n*)LGs with different *θ*_t_ (Figures 6(a) and 6(b)) imply the *θ*_t_-dependent modulation of the moiré pattern on electronic properties, moiré phonons, and the corresponding electron-phonon coupling.

## 3. Discussion

We have observed the Davydov splitting of ZO and mZO modes renormalized by PIC in MLG-MS using resonance Raman spectroscopy. The Davydov components of nonfolded ZO phonons are localized within the multilayer constituents due to the negligible perturbation from interfacial coupling, which is well represented by PM and FCM. In contrast, all the stacking layers are coupled by interfacial coupling for the vibrations of mZO phonons, and the corresponding atomic displacements show similar symmetry to the moiré pattern. The distinct difference of the vibration patterns between the Davydov components of nonfolded ZO and mZO phonon modes underpins the crucial role of PIC in the modulation of lattice dynamics for intralayer phonons in MLG-MS. This also provides potential routes to separately control the properties of nonfolded and moiré intralayer phonons. Furthermore, the rich and overlapped modulations of moiré superlattices on varied quasiparticles and the related interactions, e.g., electrons, excitons, and intralayer/interlayer phonons, will broaden the prospects for device applications of moiré materials. Remarkably, this work demonstrates that common Raman spectroscopy can provide information on the renormalized lattice dynamics in nanoscale moiré landscape of large-angle MLG-MS beyond the limit of nanotechnologies, which can be extended to other multilayer moiré superlattices. Our findings provide new insights into the rich and complex phonon physics in moiré structures, encouraging more theoretical and experimental works on this topic.

## 4. Materials and Methods

### 4.1. Sample Preparations

MLG flakes are mechanically exfoliated from the highly oriented pyrolytic graphite onto a Si/SiO_2_ wafer. During the exfoliation, an *m*-layer graphene (*m*LG) may be folded onto an *n*LG randomly and thus form the t(*m* + *n*)LG [42], such as the t(2 + 2)LG (*θ*_t_ = 16.8°) in Supplementary Figure S1(a). Alternatively, an *m*LG on one substrate can also be transferred onto an *n*LG on another substrate to form the t(*m* + *n*)LG. [43] The samples t(1 + 2)LG and t(1 + 3)LG with the same twisted angle (*θ*_t_ = 7.9°) are prepared in this way (Supplementary Figure S1(b)). After transferring, the samples were annealed in flowing H _2_/N _2_ 300  °C gas for two hours to remove the residues. In addition, the studied t(1 + 2)LGs (*θ*_t_ = 14.7°, 15.6°, and 16.4°) and t(1 + 3)LGs (*θ*_t_ = 9.5° and 14.7°) in Figure 4 were directly grown by the low-pressure chemical vapor deposition growth [44]. The layer number of MLG flakes was identified by the Raman spectra and the optical contrast (Supplementary Figure S2) [45–47]. The twist angle between the two constituents of MLG-MS is determined by comparing the characteristic mTO(R) mode frequency (below the G band) with the phonon dispersion along Γ − K direction [34]. Additionally, the twist angle is confirmed by the *θ*_t_-dependent resonance energies and mTO(R) phonon frequencies [26, 32]. Taking t(1 + 3)LG with *θ*_t_ = 11.3° as an example, Supplementary Figure S3 presents a schematic diagram for its atomic structure, which shows evident moiré pattern.

### 4.2. Raman Measurements

The Raman spectroscopy was measured under backscattering configuration at room temperature, using the Jobin-Yvon HR800 system equipped with a liquid nitrogen cooled charge-coupled (CCD) detector, a 100× objective len (NA = 0.9) and several gratings. The excitation energies (*E*_ex_) are 1.58 eV and 1.71 eV from a Ti: Sapphire laser, 1.96 eV and 2.09 eV from a He-Ne laser, 1.85 eV from a diode pumped solid-state laser, 1.83 eV, 2.18 eV, 2.34 eV, and 2.41 eV from a Kr ^+^ laser, and 2.54 eV, 2.60 eV, and 2.71 eV from an Ar ^+^ laser. The resolution at 2.60 eV is 0.07 cm^−1^ per CCD pixel. Plasma lines are removed from laser signals by using BragGrate Bandpass filters (OptiGrate Corp.). The typical laser power is ~1 mW to avoid heating. The acquisition time for each spectrum is ~1200 seconds to enhance the signal-to-noise ratio.

### 4.3. Force Constant Methods

The theoretical calculation is based on the empirical force constant method (FCM) by considering the Born-von Karman model of the lattice dynamics for atomic coupling. By solving the crystal dynamics described by

(2) $$\begin{array}{c} m_{k}{\ddot{u}}_{\alpha }\left(jk\right)=-\frac{\partial \Phi }{\partial {\ddot{u}}_{\alpha }\left(jk\right)}=-\sum \Phi_{\alpha \beta }\left(jk,j^{\prime }k^{\prime }\right)u_{\beta }\left(j^{\prime }k^{\prime }\right), \end{array}$$

the phonon dispersions of various samples, such as graphene, MLG, and t(*m* + *n*)LG can be obtained. In this equation, *m*_*k*_ is the mass of the *k*^*th*^ atom in the *j*^*th*^ unit cell; *α* and *β* enumerate the Cartesian coordinates *x*, *y*, and *z*; *u*(*jk*) specifies the displacement of the *k*^*th*^ atom in the *j*^*th*^ unit cell; and *Φ*_*αβ*_(*jk*,*j*′*k*′) is the atomic force constants that is the second derivative of the crystal potential energy *Φ* with respect to the atomic displacements taken at the equilibrium position, including the intralayer couplings and the interlayer interactions. By applying Born-von Karman model to describe the carbon-carbon intralayer interaction and taking four nearest neighbor atoms into account, the fitting parameters of the force constant can be given by the phonon dispersion of graphene. Then, the radial and tangential interlayer force constants are considered to reproduce the phonon dispersion along Γ − M direction of bulk graphite [40]. With these fitting parameters for the empirical force-constant model, we can easily access the varied phonon dispersions of t(*m* + *n*)LG and the atomic displacements of all the phonons in the BZ.

## Data Availability

All data used to support the findings of this study are available from the corresponding author upon request.

## Supplementary Materials

Supplementary Materials Figure S1: optical images of t(*m* + *n*)LGs. Figure S2: optical contrast and Raman spectra of t(1 + 3)LG. Figure S3: moiré pattern in t(*m* + *n*)LG. Figure S4: frequency differences between Davydov components of nonfolded ZO modes based on vdW model. Figure S5: Davydov splitting of nonfolded ZO modes in t(1 + 2)LG and t(2 + 2)LG. Figure S6: atomic displacements of Davydov components of mZO modes in t(1 + 2)LG. Figure S7: atomic displacements of Davydov components of the corresponding unfolded ZO modes in 3LG.

## References

[1] Bistritzer R., MacDonald A. H. (2011). Moiré bands in twisted double-layer graphene. *Proceedings of the National Academy of Sciences*.

[2] Yoo H., Engelke R., Carr S. (2019). Atomic and electronic reconstruction at the van der Waals interface in twisted bilayer graphene. *Nature Materials*.

[3] Yu H., Liu G.-B., Tang J., Xu X., Yao W. (2017). Moiré excitons: From programmable quantum emitter arrays to spin-orbit–coupled artificial lattices. *Science Advances*.

[4] Cao Y., Fatemi V., Demir A. (2018). Correlated insulator behaviour at half-filling in magic-angle graphene superlattices. *Nature*.

[5] Liu X., Hao Z., Khalaf E. (2020). Tunable spin-polarized correlated states in twisted double bilayer graphene. *Nature*.

[6] Cao Y., Fatemi V., Fang S. (2018). Unconventional superconductivity in magic-angle graphene superlattices. *Nature*.

[7] Park J. M., Cao Y., Watanabe K., Taniguchi T., Jarillo-Herrero P. (2021). Tunable strongly coupled superconductivity in magic-angle twisted trilayer graphene. *Nature*.

[8] Tran K., Moody G., Wu F. (2019). Evidence for moiré excitons in van der Waals heterostructures. *Nature*.

[9] Jin C., Regan E. C., Yan A. (2019). Observation of moiré excitons in WSe_2_/WS_2_ heterostructure superlattices. *Nature*.

[10] Seyler K. L., Rivera P., Yu H. (2019). Signatures of moiré-trapped valley excitons in MoSe_2_/WSe_2_ heterobilayers. *Nature*.

[11] Gadelha A. C., Ohlberg D. A. A., Rabelo C. (2021). Localization of lattice dynamics in low-angle twisted bilayer graphene. *Nature*.

[12] Quan J., Linhart L., Lin M.-L. (2021). Phonon renormalization in reconstructed MoS_2_ moiré superlattices. *Nature Materials*.

[13] Jin C., Kim J., Suh J. (2017). Interlayer electron-phonon coupling in WSe_2_/hBN heterostructures. *Nature Physics*.

[14] Chaudhary K., Tamagnone M., Rezaee M. (2019). Engineering phonon polaritons in van der waals heterostructures to enhance in-plane optical anisotropy. *Science Advances*.

[15] Lin M.-L., Zhou Y., Wu J.-B. (2019). Cross-dimensional electron-phonon coupling in van der Waals heterostructures. *Nature Communications*.

[16] Merkl P., Yong C. K., Liebich M. (2021). Proximity control of interlayer exciton-phonon hybridization in van der Waals heterostructures. *Nature Communications*.

[17] Tang Y., Li L., Li T. (2020). Simulation of Hubbard model physics in WSe_2_/WS_2_ moiré superlattices. *Nature*.

[18] Lin M.-L., Tan Q.-H., Wu J.-B. (2018). Moiré phonons in twisted bilayer MoS_2_. *ACS Nano*.

[19] Moore S. L., Ciccarino C. J., Halbertal D. (2021). Nanoscale lattice dynamics in hexagonal boron nitride moiré superlattices. *Nature Communications*.

[20] Chen G., Sharpe A. L., Gallagher P. (2019). Signatures of tunable superconductivity in a trilayer graphene moiré superlattice. *Nature*.

[21] Rickhaus P., de Vries F. K., Zhu J. (2021). Correlated electron-hole state in twisted double-bilayer graphene. *Science*.

[22] He M., Li Y., Cai J. (2021). Symmetry breaking in twisted double bilayer graphene. *Nature Physics*.

[23] Carozo V., Almeida C. M., Ferreira E. H. M., Cançado L. G., Achete C. A., Jorio A. (2011). Raman signature of graphene superlattices. *Nano Letters*.

[24] He R., Chung T.-F., Delaney C. (2013). Observation of low energy Raman modes in twisted bilayer graphene. *Nano Letters*.

[25] Ramnani P., Neupane M. R., Ge S., Balandin A. A., Lake R. K., Mulchandani A. (2017). Raman spectra of twisted CVD bilayer graphene. *Carbon*.

[26] Eliel G. S., Moutinho M. V., Gadelha A. C. (2018). Intralayer and interlayer electron-phonon interactions in twisted graphene heterostructures. *Nature Communications*.

[27] Parzefall P., Holler J., Scheuck M. (2021). Moiré phonons in twisted MoSe_2_–WSe_2_ heterobilayers and their correlation with interlayer excitons. *2D Materials*.

[28] Righi A., Venezuela P., Chacham H. (2013). Resonance Raman spectroscopy in twisted bilayer graphene. *Solid State Communications*.

[29] Carozo V., Almeida C. M., Fragneaud B. (2013). Resonance effects on the raman spectra of graphene superlattices. *Physical Review B*.

[30] Wu J.-B., Zhang X., Ijäs M. (2014). Resonant Raman spectroscopy of twisted multilayer graphene. *Nature communications*.

[31] DeCapua M. C., Wu Y.-C., Taniguchi T., Watanabe K., Yan J. (2021). Probing the bright exciton state in twisted bilayer graphene via resonant Raman scattering. *Applied Physics Letters*.

[32] Campos-Delgado J., Cançado L. G., Achete C. A., Jorio A., Raskin J. P. (2013). Raman scattering study of the phonon dispersion in twisted bilayer graphene. *Nano Research*.

[33] Wu J.-B., Hu Z.-X., Zhang X. (2015). Interface coupling in twisted multilayer graphene by resonant Raman spectroscopy of layer breathing modes. *ACS Nano*.

[34] Venezuela P., Lazzeri M., Mauri F. (2011). Theory of double-resonant raman spectra in graphene: intensity and line shape of defect-induced and two-phonon bands. *Physical Review B*.

[35] Wu J.-B., Lin M.-L., Cong X., Liu H.-N., Tan P.-H. (2018). Raman spectroscopy of graphene-based materials and its applications in related devices. *Chemical Society Reviews*.

[36] Song Q.-J., Tan Q.-H., Zhang X. (2016). Physical origin of davydov splitting and resonant Raman spectroscopy of davydov components in multilayer MoTe_2_. *Physical Review B*.

[37] Tan Q.-H., Zhang X., Luo X.-D., Zhang J., Tan P.-H. (2017). Layer-number dependent high-frequency vibration modes in few-layer transition metal dichalcogenides induced by interlayer couplings. *Journal of Semiconductors*.

[38] Leng Y.-C., Lin M.-L., Zhou Y. (2021). Intrinsic effect of interfacial coupling on the high-frequency intralayer modes in twisted multilayer MoTe_2_. *Nanoscale*.

[39] Mercer J. L., Chou M. Y. (1994). Tight-binding model with intra-atomic matrix elements. *Physical Review B*.

[40] Wang H., Wang Y. F., Cao X. W., Feng M., Lan G. X. (2009). Vibrational properties of graphene and graphene layers. *Journal of Raman Specroscopy*.

[41] Cocemasov A. I., Nika D. L., Balandin A. A. (2013). Phonons in twisted bilayer graphene. *Physical Review B*.

[42] Novoselov K. S., Jiang D., Schedin F. (2005). Two-dimensional atomic crystals. *Proceedings of the National Academy of Sciences*.

[43] Dean C. R., Young A. F., Meric I. (2010). Boron nitride substrates for high-quality graphene electronics. *Nature Nanotechnology*.

[44] Song Y., Pan D., Cheng Y., Wang P., Zhao P., Wang H. (2015). Growth of large graphene single crystal inside a restricted chamber by chemical vapor deposition. *Carbon*.

[45] Ferrari A. C., Meyer J. C., Scardaci V. (2006). Raman spectrum of graphene and graphene layers. *Physical Review Letters*.

[46] Zhao W., Tan P., Zhang J., Liu J. (2010). Charge transfer and optical phonon mixing in few-layer graphene chemically doped with sulfuric acid. *Physical Review B*.

[47] Li X.-L., Han W.-P., Wu J.-B., Qiao X.-F., Zhang J., Tan P.-H. (2017). Layer-number dependent optical properties of 2D materials and their application for thickness determination. *Advanced Functional Materials*.

