# Supplementary material for: Intralayer Phonons in Multilayer Graphene Moiré Superlattices
Source: Research (Wash D C). 2022 May 30;2022:9819373. doi: 10.34133/2022/9819373 (PMC9175117; doi:10.34133/2022/9819373)
Supplement: Supplementary Materials — Figure S1: optical images of t(m + n)LGs. Figure S2: optical contrast and Raman spectra of t(1 + 3)LG. Figure S3: moiré pattern in t(m + n)LG. Figure S4: frequency differences between Davydov components of nonfolded ZO modes based on vdW model. Figure S5: Davydov splitting of nonfolded ZO modes in t(1 + 2)LG and t(2 + 2)LG. Figure S6: atomic displacements of Davydov components of mZO modes in t(1 + 2)LG. Figure S7: atomic displacements of Davydov components of the corresponding unfolded ZO modes in 3LG. [file 9819373.f1.pdf]

Supplementary Materials for Intralayer phonons in multilayer graphene moiré superlattices

Miao-Ling Lin[1,2], Min Feng[3], Jiang-Bin Wu[1], Fei-Rong Ran[4], Tao Chen[1], Wei-Xia Luo[1,2], Heng Wu[1,2], Wen-Peng Han[1], Xin Zhang[1], Xue-Lu Liu[1], Yang Xu[5], Hai Li[4], Yu-Fang Wang[3], and Ping-Heng Tan[1,2*]

[1]State Key Laboratory of Superlattices and Microstructures, Institute of Semiconductors, Chinese Academy of Sciences, Beijing 100083, China

[2]Center of Materials Science and Optoelectronics Engineering & CAS Center of Excellence in Topological Quantum Computation, University of Chinese Academy of Sciences, Beijing 100049, China

[3]School of Physics, Nankai University, Tianjin 300071, China

[4]Key Laboratory of Flexible Electronics and Institute of Advanced Materials, Nanjing Tech University, 30 South Puzhu Road, Nanjing 211816, China

[5]State Key Laboratory of Silicon Materials, College of Material Science, Zhejiang University, Hangzhou 310027, China

[*]Corresponding author. Email: phtan@semi.ac.cn

Abstract

The contents of the Supplementary Materials are summarized as follows: (1) optical images of multilayer graphene moiré superlattice (MLG-MS) and the lattice structures; (2) comparison of frequency difference of Davydov components between vdW model and experimental results; (3) Davydov splitting of nonfolded ZO modes in t(1+2)LG and t(2+2)LG; (4) the vibration patterns for each Davydov component of mZO modes in t$(m+n)$LGs.

1 Optical images of MLG-MS and the lattice structures

Here we show the typical optical images and the schematic lattice structures of MLG-MS. The layer number of MLG flakes is identified by optical contrast and Raman spectra.

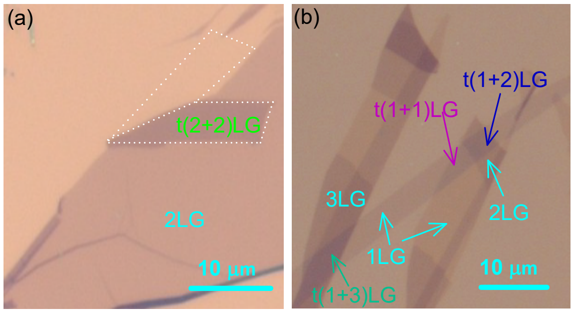

Figure 1: Optical images of t($m+n$)LGs. Optical images of (a) t(2+2)LG with twist angle $\theta_{\rm t}$=16.8° (b) t(1+2)LG and t(1+3)LG twist angle $\theta_{\rm t}$=7.9°. (a) is prepared by self-folding during the exfoliation while (b) is produced by transferring. The scale bars are shown.

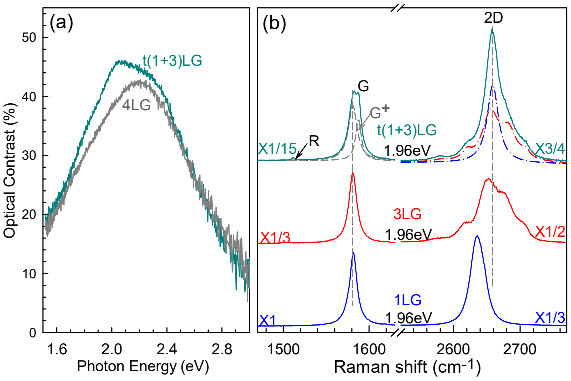

Figure 2: Optical contrast and Raman spectra of t(1+3)LG. (a) Optical contrast of t(1+3)LG and 4LG. (b) Raman spectra of t(1+3)LG, 1LG and 3LG in the G and 2D spectral range.

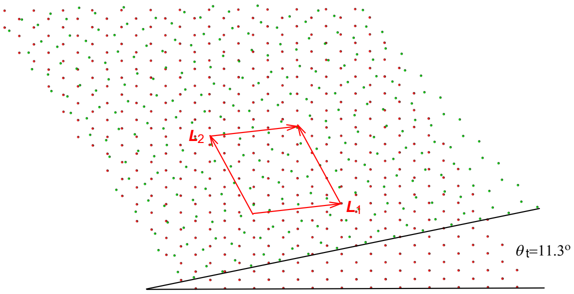

Figure 3: Moiré pattern in t($m+n$)LG. Schematic structure of t(1+3)LG with the 1LG (green) sitting on the top of the 3LG (red). The 1LG and 3LG constituents are twisted by an angle ($\theta_{\rm t}$) of 11.3°, which shows evident moiré pattern. Vectors $\boldsymbol{L}_1$ and $\boldsymbol{L}_2$ define the supercell.

2 Comparison of frequency difference of Davydov components between vdW model and experimental results

The Davydov splitting in multilayer and twisted transition metal dichalcogenides (TMDs) are well represented by the vdW model by considering the interlayer coupling as the first approximation,

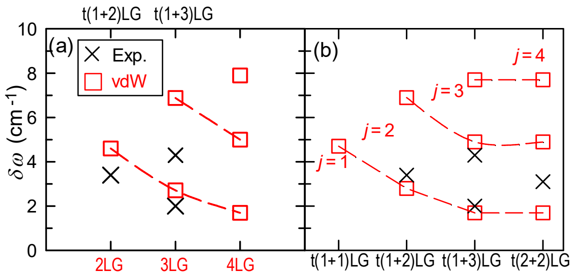

Figure 4: Frequency differences between Davydov components of nonfolded ZO modes based on vdW model. The experimental (black crosses) and calculated (open circles) frequency differences ($\Delta\omega$) between each Davydov components and the lowest-frequency one in t$(m+n)$LGs without (a) and with (b) interfacial coupling considered.

where the frequency differences between each Davydov component and the lowest-frequency one can be estimated by the frequency of the corresponding layer-breathing phonon with the same number of in-phase/out-of-phase interlayer vibrations between the nearest adjacent atomic planes [1–3].Here we tried to apply the vdW model to understand the observed Davydov components in MLG-MS. Firstly, we neglect interfacial coupling and apply the vdW model to the calculate the frequency difference between the Davydov components and the uncoupled (lowest-frequency) one in mLG and nLG constituents. We predict the Davydov components in t(1+2)LG and t(1+3)LG are respectively the same as those in 2LG and 3LG. Indeed, the number of the observed Davydov components is in line with the prediction. However, clear deviations of frequency differences are present in Figure 4(a). Considering the interfacial coupling is close to the interlayer coupling in AB-stacked MLG, the corresponding Davydov components can be also calculated. In this case, both the number of Davydov components and frequency differences between each Davydov component and the lowest-frequency one are inconsistent with the experimental results, as elucidated in Figure 4(b). This suggests that the vdW model commonly used to reproduce the Davydov splitting in TMDs consisting of three-atomic layers is not applicable for MLG-MS.

3 Davydov splitting of nonfolded ZO modes in t(1+2)LG and t(2+2)LG

According to the PM, the Davydov splitting of nonfolded ZO phonon in t(2+2)LG is localized within the 2LG constituents, as shown in Figure 5(a). This is the same as in t(1+2)LG (Figure 5(b)) and consistent with the observation in experiment, further confirms the validity of PM to understand the localized Davydov components of nonfolded ZO phonons within the constituents of t$(m+n)$LGs, including the assumption of negligible perturbation from patterned interfacial coupling (i.e., ϵ_t=0).

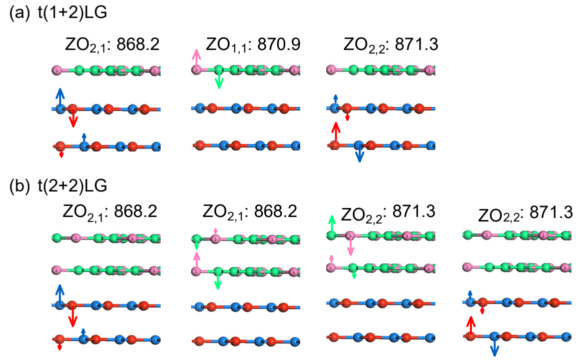

Figure 5: Davydov splitting of nonfolded ZO modes in t(1+2)LG and t(2+2)LG. Atomic displacements and mode frequencies of Davydov components for nonfolded ZO phonons in (a) t(1+2)LG and (b) t(2+2)LG by the SPM, the arrow lengths represent the amplitudes of atomic displacements.

4 The vibration patterns for each Davydov component of mZO modes in t$(m+n)$LGs

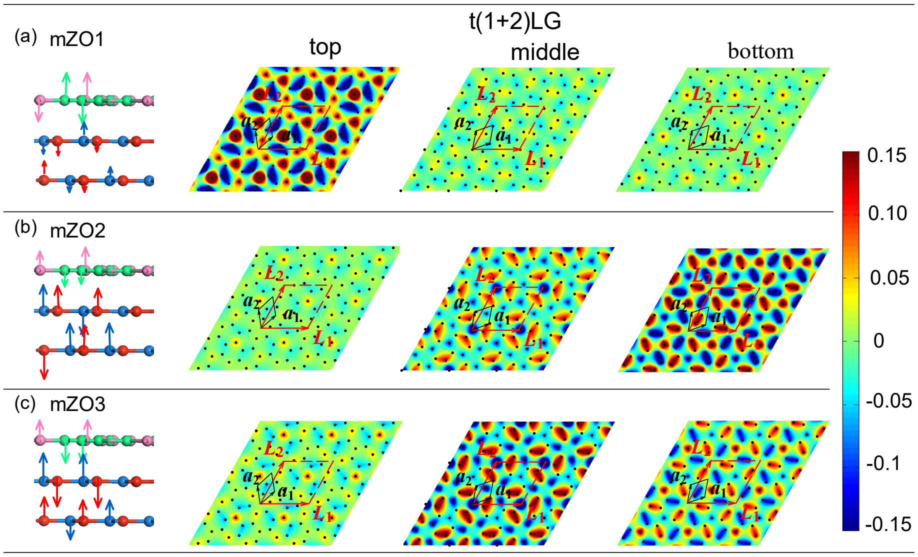

Figure 6: Atomic displacements of Davydov components of mZO modes in t(1+2)LG. Atomic displacements of three Davydov components of mZO in a 3×3 supercell of t(1+2)LG with θ_t=21.8°: (a) mZO$_1$ (b) mZO$_2$ and (c) mZO$_3$.

The modulations from moiré superlattices can be directly reflected by the corresponding atomic displacements of each Davydov component of mZO modes. In contrast to nonfolded ZO phonon where the interfacial coupling is really weak to couple three layers, the patterned interfacial coupling introduces a perturbation to the mZO phonons and leads to three Davydov components, as shown in Figure 6. Furthermore, the vibrations for mZO phonons in t(1+2)LG are modulated by the moiré superlattices and the vibrations at each layer show high symmetry. However, the atomic

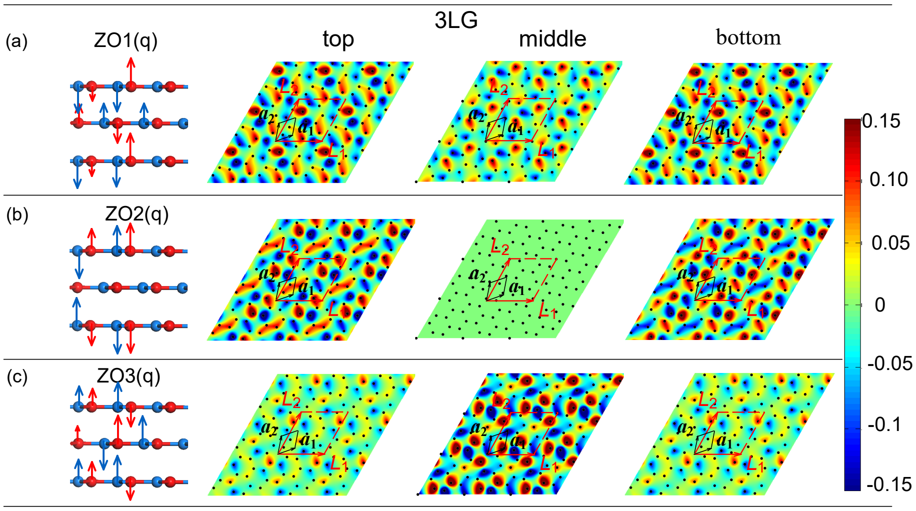

Figure 7: Atomic displacements of Davydov components of the corresponding unfolded ZO modes in 2LG. Atomic displacements of three Davydov components of the corresponding unfolded ZO(q) in AB-stacked 3LG: (a) $\mathrm{ZO}_1(q)$ (b) $\mathrm{ZO}_2(q)$ and (c) $\mathrm{ZO}_3(q)$, where $\boldsymbol{q}$ is the wave vector same as the moiré reciprocal basic vector.

displacements of the corresponding unfolded $\mathrm{ZO}_i(q)$ $(i = 1, 2, 3)$ phonon in 3LG exhibit distinctly different vibration patterns (Figure 7), without any signature of C_3 symmetry.

References

[1] Q.-J. Song, Q.-H. Tan, X. Zhang, J.-B. Wu, B. W. Sheng, Y. Wan, X.-Q. Wang, L. Dai, and P.-H. Tan, "Physical origin of davydov splitting and resonant raman spectroscopy of davydov components in multilayer mote$_2$," *Phys. Rev. B*, vol. 93, p. 115 409, 11 2016.

[2] Q.-H. Tan, X. Zhang, X.-D. Luo, J. Zhang, and P.-H. Tan, "Layer-number dependent high-frequency vibration modes in few-layer transition metal dichalcogenides induced by interlayer couplings," *J. Semicond.*, vol. 38, no. 3, p. 031 006, 2017.

[3] Y.-C. Leng, M.-L. Lin, Y. Zhou, J.-B. Wu, D. Meng, X. Cong, H. Li, and P.-H. Tan, "Intrinsic effect of interfacial coupling on the high-frequency intralayer modes in twisted multilayer mote$_2$," *Nanoscale*, vol. 13, pp. 9732–9739, 21 2021.
